# Cognitive and hallucinatory-paranoid disorders in vascular dementia (clinical and psychopathological structure and diagnostic criteria)

**DOI:** 10.1192/j.eurpsy.2026.10443

**Published:** 2026-07-31

**Authors:** V. Kosovskyi

**Affiliations:** Department of Psychiatry, Addiction Medicine and Medical Psychology, I. Horbachevsky Ternopil National Medical University, Ternopil, Ukraine

## Abstract

**Introduction:**

Dementia is a severe neurodegenerative disorder, the mechanisms of formation of which are closely related to age, which allowed to classify this pathology as an age-related disease. According to the results of modern research, more than 50 million people in the world have dementia and their number is growing annually, and in 2050 it will reach 152.8 (130.8 to 175.6) million cases, which is associated with population growth and aging

**Objectives:**

The aim of the research is to study their clinical and psychopathological structure, mutual influence and develop their diagnostic criteria based on a comprehensive assessment of cognitive and hallucinatory-paranoid disorders in patients with vascular dementia (at different stages of the pathological process)

**Methods:**

The work was based on the results of the study of 75patients with vascular dementia (VD) and hallucinatory-paranoid disorders (HPD), who made up the main group. As a control group, 63 patients with VD without predominant HPD participated in the study. The complex of research methods included clinical and psychopathological, psychometric, psychodiagnostic and mathematical statistical methods

**Results:**

The results of the study showed that the characteristics of HPD and cognitive disorders (CD) depend on the stage of VD development. The clinical and psychopathological structure of HPD in patients with VD in the middle stage of development (MSD) was characterized by the predominance of paranoid disorders with systematic ideas of damage, robbery, theft, which occurred in the form of paranoid delusional disorder.

**Conclusions:**

The study of the clinical and psychopathological structure of HPD in patients with VD revealed its dependence on the stage of development of the pathological process.In patients with VD in the MSD, frequent paranoid disorders with a systematized delusional plot of material damage, robbery, theft, relationships and jealousy of moderate severity, which occurred in the form of paranoid delusional disorder, acute paranoia and hallucinosis, dominated the structure of clinical manifestations.

**Disclosure of Interest:**

None Declared

